# Multidimensional poverty indicators in Brazil’s Legal Amazon

**DOI:** 10.1590/0102-311XEN212223

**Published:** 2025-03-24

**Authors:** Camila de Moura Vogt, Ricardo Bruno Nascimento dos Santos, Danilo Araújo Fernandes

**Affiliations:** 1 Universidade Federal de Santa Maria, Santa Maria, Brasil.; 2 Universidade Federal do Pará, Belém, Brasil.

**Keywords:** Amazonian Ecosystem, Poverty, Social Indicators, Income, Ecosistema Amazónico, Pobreza, Indicadores Sociales, Renta

## Abstract

In public health research, socioeconomic indicators play a crucial role. They are essential for understanding the relation between poverty and health issues, as well as for allocating resources to public health infrastructure and providing appropriate treatments to the population. Traditionally, income has been the main indicator for measuring poverty, by establishing a poverty line that distinguishes those considered not poor or not vulnerable (above the line) from those categorized as poor or vulnerable (below the line). The one-dimensional analysis of poverty, however, shows limitations, particularly in regions with high epidemiological complexity and highly vulnerable populations, such as Brazil’s Legal Amazon. This paper discusses and estimates a multidimensional approach to poverty in Brazil’s Legal Amazon based on the multidimensional poverty index (MPI) according to the Alkire-Foster (2010) methodology. As a socioeconomic indicator, the MPI considers not only income, but also other indicators related to public health that affect the well-being of populations. The use of a multidimensional indicator allows a more complete understanding of socioeconomic and living conditions, facilitating the formulation of public policies. The results of the calculated indicator, MPI - Legal Amazon, show important differences in the characteristics of rural poverty, as well as in the intensity and incidence of this poverty, which are not identified by one-dimensional income indicators. The paper concludes that the multidimensional approach is key to address the complex issues of public health and socioeconomic development in the region.

## Abstract (es)

Los indicadores socioeconómicos juegan un papel fundamental en los estudios de salud pública. Son esenciales para comprender la relación entre la pobreza y los problemas de salud, así como para conducir la asignación de recursos en la infraestructura de salud pública y en la provisión de tratamientos adecuados a la población. Los ingresos han sido el principal indicador utilizado para medir la pobreza, estableciendo un umbral de pobreza que determina quién está por encima de él (clasificado como no pobre o no vulnerable) y quién está por debajo (considerado pobre o vulnerable). Sin embargo, el análisis unidimensional de la pobreza es limitado, particularmente en regiones con alta complejidad epidemiológica y poblaciones muy vulnerables, como la Amazonía Legal. Este estudio discute y calcula un enfoque multidimensional de la pobreza en la Amazonía Legal basado en el índice de pobreza multidimensional (IPM) de acuerdo con la metodología Alkire-Foster (2010). El IPM considera no solo los ingresos como indicador socioeconómico, sino también otros indicadores relacionados con la salud pública que afectan el bienestar de las poblaciones. El uso de un indicador multidimensional favorece una comprensión más completa de las condiciones socioeconómicas y de vida, facilitando la formulación de políticas públicas. Los resultados del indicador calculado, IPM - Amazonía Legal, muestran diferencias significativas en las características de la pobreza rural, así como en la intensidad e incidencia de la pobreza, que no son identificadas por los indicadores unidimensionales de renta. Se concluye que un enfoque multidimensional es fundamental para abordar los complejos problemas de salud pública y de desarrollo socioeconómico en la región.

## Introduction

Socioeconomic indicators are extremely important for public health studies, especially in areas with high epidemiological complexity and highly vulnerable populations, as is the case of Brazil’s Legal Amazon ^1^ ^,^ ^2^ ^,^ ^3^ ^,^ ^4^. Variables that can measure poverty and socioeconomic vulnerabilities are directly related to the spread of diseases, mortality, public health infrastructure, and types of treatments that will be offered to the population. The characterization of poverty and vulnerabilities, however, is a controversial topic for the public policy debate. The means to define a poor or socioeconomically vulnerable person is not static; it can vary over time and across cultures. Traditionally, poverty is measured by income and is linked to individuals’ capacity to consume.

The distinction between poverty and vulnerability, however, is crucial to understand the complexities of socioeconomic conditions and the needs of deprived populations. Poverty refers to the scarcity of basic material resources, such as income, food, and housing, whereas vulnerability encompasses exposure to risks and the capacity to cope with or recover from them. Although poverty can be a contributing factor to vulnerability, the latter is influenced by many other aspects, such as social exclusion, discrimination, lack of access to essential services, and adverse events like natural disasters and armed conflicts. Understanding this distinction allows for the development of more effective policies and interventions to address the specific needs of vulnerable communities ^5^ ^,^ ^6^ ^,^ ^7^ ^,^ ^8^ ^,^ ^9^ ^,^ ^10^.

The most common method of measuring poverty is by establishing a poverty line, which is a monetary threshold that distinguishes those above it as not poor or not vulnerable, and those below it as poor or vulnerable. This methodological choice is usually justified by the convenience of data or the capacity that monetary limitation holds to define a population’s well-being. After all, the inability to acquire a certain basket of goods can reveal much about the level of vulnerability that a family or individual faces ^9^ ^,^ ^11^ ^,^ ^12^ ^,^ ^13^.

The one-dimensional analysis of the situation of poverty, however, is not a consensus to express human development. Despite the relevance of income, other factors can be associated with poverty. Access to public services, healthcare, education, employment, and safety are strongly linked to well-being but are not fully explained by a population’s capacity to consume. Similarly, there are differences between poverty levels and the types of public policies needed for each specific population, which are underidentified in one-dimensional measurement methods ^11^.

Brazil’s Legal Amazon comprises an extensive portion of the Amazon, which is the largest tropical forest in the world, located within Brazilian territory. This region covers approximately 58.93% of Brazil’s territory, with a total area of 5,015,146.008km^2^. The term Legal Amazon is based on legal and administrative criteria, encompassing nine Brazilian states, namely: Acre, Amapá, Amazonas, Maranhão, Mato Grosso, Pará, Rondônia, Roraima, and Tocantins, covering a total of 772 municipalities ^14^. Throughout history, the Amazon has often been perceived in a dualistic view. On the one hand, it is seen as a region marked by poverty, vulnerability, and backwardness; on the other hand, it is considered rich due to its potential for exploiting natural resources. This dualistic approach, which separates the social and economic dimensions from the environmental reality, has hindered the understanding on the various ways in which local populations use biodiversity and how this affects the well-being and vulnerabilities of populations.

Given the complexities of the Brazilian Legal Amazon, the use of socioeconomic indicators that address all the characteristics of the region is a challenging task. In this aspect, the choice of socioeconomic indicators can lead to misconceptions and incorrect evaluations. This study aims to assess the multidimensional indices used to identify poverty and to emphasize the multidimensional poverty index (MPI) in the Legal Amazon. The MPI is an indicator that holds several methodological advantages for understanding the socioeconomic situation of the region. To this end, the MPI - Brazilian Legal Amazon was estimated, and the ways in which this indicator can transcend the purely monetary approach in evaluating public policies were explored. This is due to its flexible methodology, which considers different family characteristics and integrates them into a single index. This approach enables a more comprehensive and detailed analysis of the living conditions of local populations, yielding a more accurate understanding of the needs and challenges faced by the Amazon Region.

Considering this issue, multidimensional approaches have gained prominence for the assessment of poverty. Unlike one-dimensional approaches that traditionally limit themselves to a single indicator, multidimensional approaches includes various aspects of people’s lives, which has allowed a better assessment of the influence of its components linked to the specificity of a given territory, resulting in a more complete understanding of the deprivations within such territory. These multidimensional approaches can be classified into three main categories: axiomatic, as proposed by Alkire & Santos ^15^ ^),^ and information theory; fuzzy methodologies; and latent variable theories,.

Among the multidimensional approaches, the Dashboard methods ^16^ and composite indices are widely used, especially in the context of human development, as described by Alkire & Santos ^(^ ^15^ and Alkire et al. ^17^. A notable example is the United Nations Sustainable Development Goals (SDGs) program, which uses a Dashboard consisting of 49 indicators to monitor 17 goals and 169 targets ^18^ ^,^ ^19^ ^,^ ^20^. In Brazil, the Dashboard method has been largely employed; however, it has struggled to maintain temporal rigor concerning the available databases and comparisons between territories, especially between metropolitan areas (with higher microdata availability) and non-metropolitan areas (with limited microdata) ^19^.

The Human Development Index (HDI) ^21^ and the human poverty index (HPI) ^22^ are examples of composite indices that incorporate multiple dimensions related to poverty. However, despite their wide use, these indices are limited regarding identification of simultaneous deprivations and characterization of spatial heterogeneities, especially the differences between rural and urban areas.

Other multidimensional indicators, which assess social vulnerabilities more broadly, are also used for the Amazon Region. The social progress index (SPI) is a comprehensive measure that evaluates the social and environmental performance of nations without relying solely on economic development. Created in 2013 by the Social Progress Imperative, the SPI recognizes that economic growth alone does not guarantee social progress and can lead to problems such as exclusion, social discontent, and environmental degradation ^23^. In its 2018 structure, the index included 51 social and environmental indicators distributed in three dimensions (Basic human needs, Foundations of wellbeing, and Opportunity) and 12 components, based on public data available online.

Despite being widely used and easy to measure due to working with national, state, and municipal aggregates, multidimensional methodologies such as SDGs, HDI, HPI, and SPI fail to identify simultaneous deprivations and adequately characterize spatial heterogeneities, particularly rural and urban differences. Thus, they are not the most appropriate methods for measuring the specific vulnerabilities of Brazilian households. Axiomatic measures, such as the MPI, show several convenient characteristics. First, they fulfill the two steps necessary for measuring poverty: identifying the poor and aggregating the information into a single indicator. Second, the portfolio of axiomatic measures includes measures that only apply when the indicators are cardinal, but also includes measures that apply when the indicators are ordinal ^17^ ^,^ ^24^. Additionally, the MPI - despite being a synthetic indicator - allows for the analysis of each dimension that characterizes the understanding of poverty and vulnerability separately, making it possible to understand the weight of each dimension in defining poverty.

The estimation of the MPI requires the identification of the units of analysis, typically households, and the assessment of the deprivations they face in various dimensions. Units are considered multidimensionally poor when their deprivation score crosses an interdimensional threshold. The Global MPI, based on the Alkire-Foster method, is used on a global scale and incorporates five dimensions of well-being: income poverty, education, access to basic infrastructure, health and nutrition, and safety ^25^.

In the Brazilian scenario, several academic studies have applied MPI and other multidimensional approaches to assess the living conditions and well-being of the population. These studies are based on microdata from national surveys, such as the various Brazilian censuses and the *Brazilian National Household Sample Survey* (PNAD, acronym in Portuguese). Academic research in Brazil has addressed a variety of dimensions, including health, education, living standards, safety, and access to basic services, in both urban and rural areas. These studies play a fundamental role in the analysis of the living conditions of the Brazilian population and in the identification of regional inequalities. They provide valuable information for the development of public policies aimed at improving the quality of life throughout the country. Therefore, multidimensional approaches have proven to be important instruments to understand the complexity of poverty and to guide government actions aimed at promoting well-being and combating inequality.

As outlined by Santos et. al ^26^, within the scope of Brazilian academic research, several studies address the assessment of quality of life and well-being in different regions of the country, employing a variety of dimensions and indicators. Bagolin & Ávila ^27^ have investigated at national level, the dimensions related to health, safety, education, and food security, using indicators such as hospital beds, number of physicians, infant mortality, life expectancy, homicides, number of police officers, illiteracy, schooling, and proportion of people with food restrictions. Barros et al. ^28^ have focused on dimensions of vulnerability, access to knowledge, access to work, resource scarcity, child development, and housing deprivation, comparing rural and urban areas at the national level. Its indicators include fertility, special attention and care for different age groups, demographic dependence, and maternal presence, among others.

Several other recent studies have achieved important results in demonstrating the complexity of the impacts of different dimensions and specificities of poverty and vulnerability in different regions of Brazil ^29^ ^,^ ^30^ ^,^ ^31^ ^,^ ^32^ ^,^ ^33^ ^,^ ^34^ ^,^ ^35^ ^,^ ^36^.

For the Brazilian Legal Amazon, the choice of indicators that address poverty and socioeconomic characteristics in a multidimensional way is even more pertinent. Intra-regional economic inequalities in the Brazil’s Legal Amazon are significant. While some areas can benefit, directly or indirectly, from improved infrastructure and job creation in certain economic sectors considered more dynamic (such as mining and agricultural development), others suffer from a complete lack of infrastructure and access to basic services. These disparities can be observed in unequal access to health, education, employment, and security services, for example.

On the other hand, many regions benefiting from the dynamism of economic sectors face accelerated transformations in access to natural resources crucial for ensuring food security and sustaining the livelihoods of traditional populations who rely on common areas, rivers, lakes, and forest resources to survive. Moreover, many areas of the Legal Amazon lack basic infrastructure, such as roads and electricity, which hinders access to services and economic opportunities. The resulting isolation creates a cycle of disadvantage, preventing access to markets and employment opportunities.

Strategies for development and reduction of vulnerabilities for the Amazonian population combine a series of initiatives that diverge sharply in relation to socioeconomic models and indicators. The “Deforestation Arc”, known for encompassing parts of the states of Maranhão, Tocantins, Pará, Mato Grosso, Rondônia, and Acre, is considered one of the active frontiers of land use regarding deforestation and wildfires. Despite this, this region shows high indicators of gross domestic product (GDP) per capita and HDI. The controversy between economic growth and deforestation is recurrent in the debate about a model of growth and development throughout the Amazon Region.

According to Guedes et al. ^38^, in a study conducted in the region of Altamira in Pará, the multidimensional conditions of well-being reveal that some economically viable land use strategies by small landowners (e.g., pastures) can have significant ecological implications for the regional landscape. This ultimately affects the guarantee and right of access to goods and services that are used and collectively beneficial to a large population in the region. Other studies on the Legal Amazon - using multidimensional indicators - elucidate the complex relationship between structural issues in the region and factors that go beyond per capita income indicators ^4^ ^,^ ^26^ ^,^ ^36^ ^,^ ^37^.

## Methodology

The Alkire-Foster MPI method ^24^ can be segmented into two phases: identification and aggregation. In an intuitive way, these phases can be separated into stages; in the identification phase six steps are considered. The first step consists of defining the set of indicators that will be considered in the multidimensional measure, considering that the data for the indicators need to be available for the same person or family observed. In the second and third step, the cut-off points for each indicator and the definition of deprivations are defined. Finally, in the last three step of identification, the weight or relative value that each indicator holds is selected, so that they add up to one. Thus, the “deprivation score” is created, and the poverty threshold is normatively determined, which corresponds to the proportion of weighted deprivations a person must face to be considered multidimensionally poor.

In the aggregation phase, seven additional steps are considered. First, the estimation of the proportion of people who have been identified as multidimensionally poor in the population. This is the multidimensional poverty index *H*, also called the incidence of multidimensional poverty. Next, the intensity of multidimensional poverty *A* is calculated, or the breadth of poverty, which is the average proportion of deprivations. The *M*_*0*_ , *M*_*1*_ , and *M*_*2*_ indices are also estimated, considering only the populations identified as poor. In which *M*_*0*_ is the adjusted index, typically used as an indicator, and *M*_*0*_ = H * A is the sum of weighted deprivations experienced by the poor divided by the total population. Meanwhile, *M*_*1*_ considers the average shortfall in poverty in all cases in which poor people are deprived, or the depth of poverty *G*. Being *M*_*1*_ = H * A * G, or the sum of differences in deprivation divided by the total population. Finally, indicator *M*_*2*_ considers the severity of poverty, *S*, which is the average of the deprivation differences squared. Thus, *M*_*2*_ = H * A * S, or the sum of the deprivation gaps squared, divided by the total population.

The index represents the number of deprivations the individual suffers at any given time. Thus, individuals considered poor do not necessarily experience all, but a set of *i* basic deprivations *c*_*i*_ overlapping. The variable *k* is the poverty indicator threshold and reflects the weighted sum of indicators at which an individual is considered multidimensionally poor. Thus, the MPI is the multiplication of two terms: *H*, the poverty incidence, $H=\frac{q}{n}$ , in which *q* is the number of multidimensionally poor individuals and *n* is the total population; and *A*, the poverty intensity (Equation 1) ^24^.

$$A=\frac{1}{q}\sum_{i=1}^{q}c_{i}\left(k\right)$$ (Equation 1)

In this way, the MPI indicator can also be described according to Equation 2:

$$MPI=\frac{1}{n}\sum_{i=1}^{n}c_{i}\left(k\right)$$ (Equation 2)

The maximum count of deprivation is 100%, and in the MPI - Brazilian Legal Amazon, four dimensions were considered, in which each dimension received a weighting of 1/4. Thus, the maximum count in each dimension or the poverty cut-off point is 25%. Each indicator was weighted by dividing the weighting (1/4) by the number of indicators. The cut-off point in the MPI - Brazilian Legal Amazon was defined in proportion to the number of dimensions. To identify multidimensionally poor households, the needs of each dimension were added to obtain the corresponding total need of each household.

The database comes from household microdata based on the 2000 and 2010 Brazilian Demographic Censuses ^39^ ^,^ ^40^. The data covers all municipalities in Brazil’s Legal Amazon, encompassing nine Brazilian states and a total of 756 out of 772 municipalities in the region (municipalities created from 2000 to 2010 and municipalities without information for this period were excluded from the analysis) ^39^ ^,^ ^40^. All information was generated according to the Alkire-Foster method for the estimation of the municipal MPI of the Legal Amazon.

Box 1 highlights the variables used and their dimension.

**Box 1:** Description of the variables, dimensions, and weights used in the multidimensional poverty index (MPI).

VARIABLE	DIMENSION	INDICATOR WEIGHT IN MPI
Newborn mortality	Health	1/4
Adults without primary education	Education	1/12
Head of the household is illiterate	Education	1/12
Out-of-school children	Education	1/12
No sanitary sewer	Living standards	1/28
Garbage without proper disposal	Living standards	1/28
No electricity	Living standards	1/28
No access to piped water	Living standards	1/28
Lives in an improvised or collective home	Living standards	1/28
Household density of more than 5 people	Living standards	1/28
Leased land	Living standards	1/28
Income from social programs only	Employment conditions and private assets	1/20
Head of household without paid work	Employment conditions and private assets	1/20
No refrigerator	Employment conditions and private assets	1/20
People with no access to digital technology	Employment conditions and private assets	1/20
People with less than two private assets	Employment conditions and private zssets	1/20

Source: prepared by the authors based on the 2000 and 2010 Brazilian Demographic Censuses ^39^ ^,^ ^40^.

The MPI - Brazilian Legal Amazon, thus, was composed of 16 indicators for the rural and urban settings in four different dimensions. The set of indicators was selected from recent studies4, according to the information collected in the sample questionnaire for the aforementioned census survey.

## Results

The results of the MPI - Brazilian Legal Amazon can be divided and analyzed considering specific issues such as urban and rural predominance, territorial issues, or specific segregations considering households, census regions, municipalities, states, and finally the Legal Amazon Region as a whole. Notably, the outcomes are limited to analyze the evolution of the different regions for the municipalities and states over the years.

**Figure 1:**
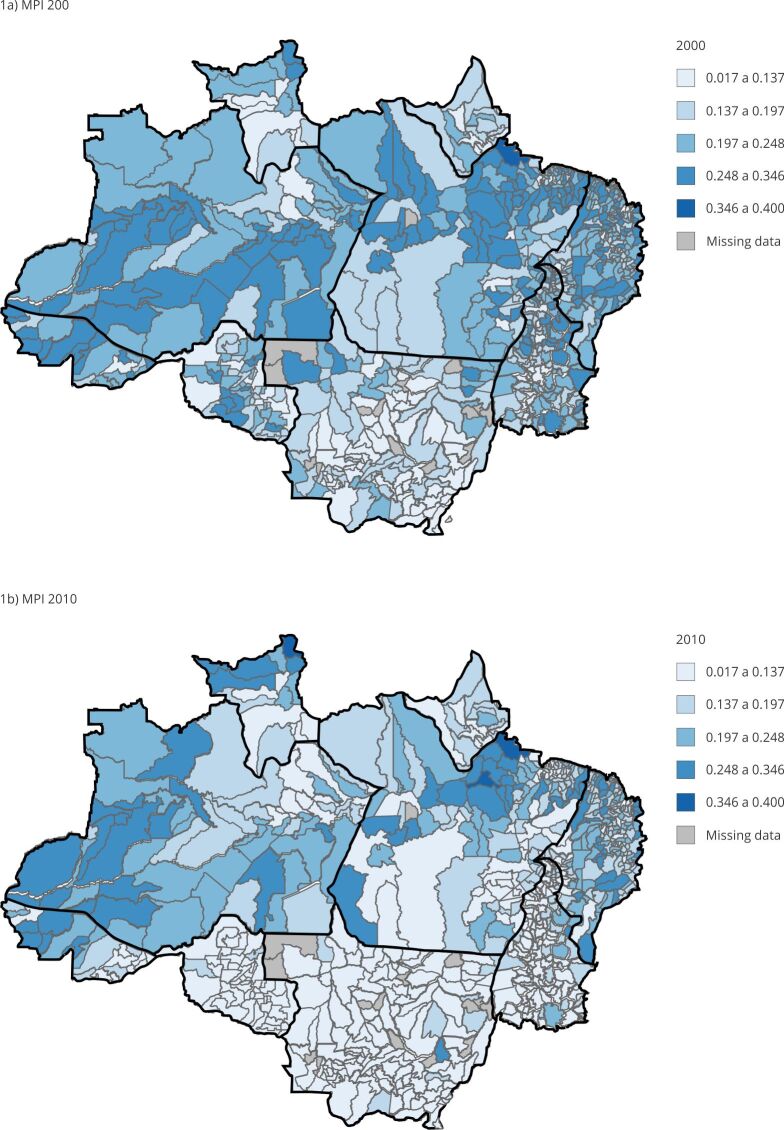
Spatial evolution of multidimensional poverty index (MPI) from 2000 to 2010. Legal Amazon, Brazil.

Source: prepared by the authors based on the 2000 and 2010 Brazilian Demographic Censuses ^39^ ^,^ ^40^.

**Figure 2:**
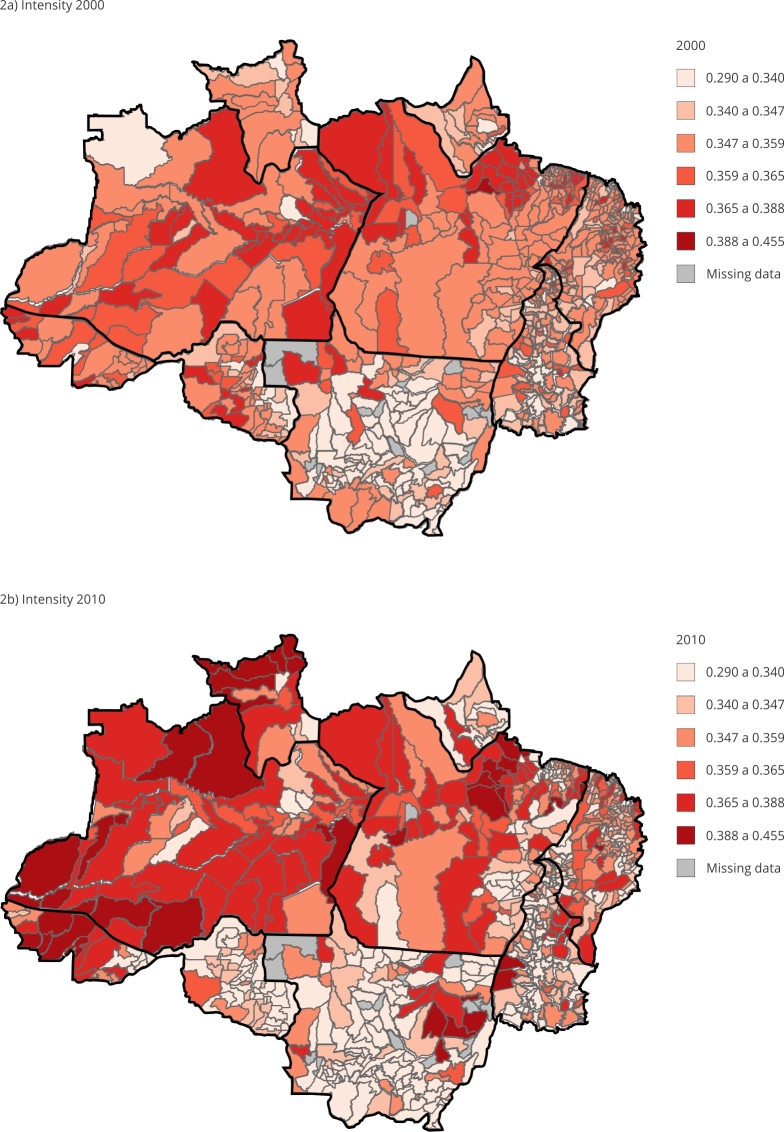
Spatial evolution of intensity of multidimensional poverty (A) from 2000 to 2010. Legal Amazon, Brazil.

Source: prepared by the authors based on the 2000 and 2010 Brazilian Demographic Censuses ^39^ ^,^ ^40^.

**Figure 3:**
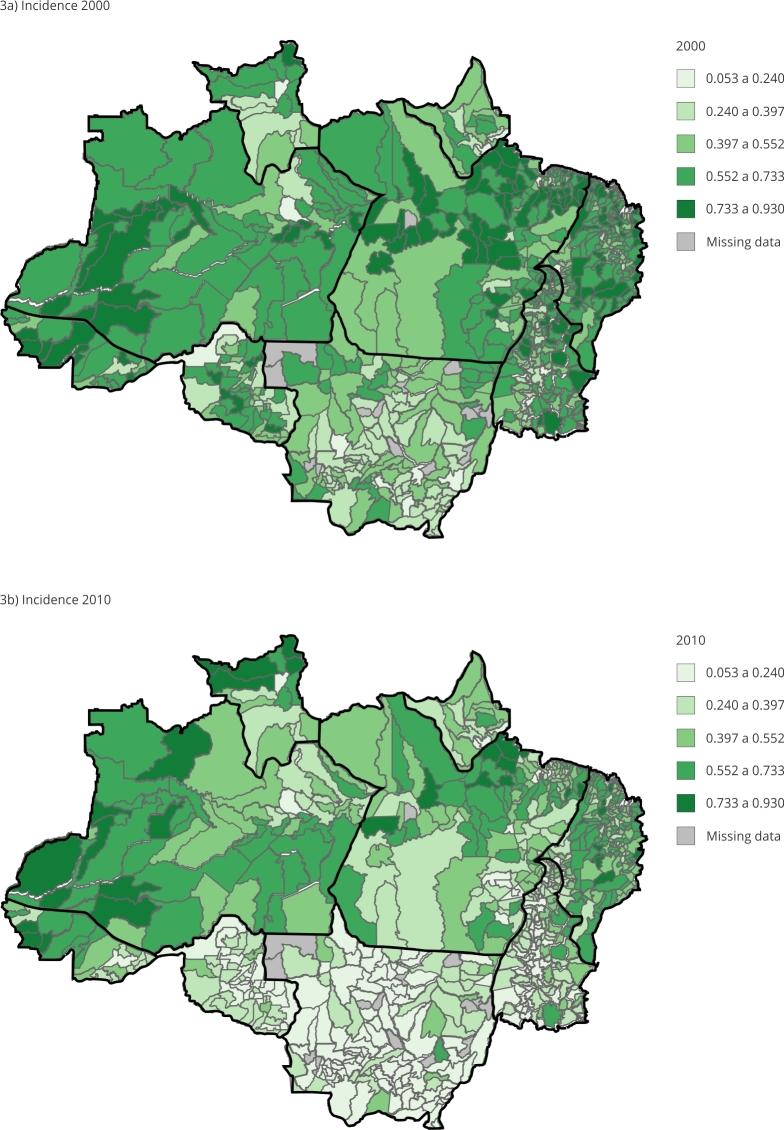
Spatial evolution of incidence of multidimensional poverty (H) from 2000 to 2010.

Source: prepared by the authors based on the 2000 and 2010 Brazilian Demographic Censuses ^39^ ^,^ ^40^.

Figures 1, 2 and 3 show the MPI and the two components that constitute incidence (H) and intensity (A) from 2000 to 2010 for predominantly urban and rural areas according to the Brazilian Institute of Geography and Statistics (IBGE) classification of municipalities in the Legal Amazon. By verifying their variation profile over time, the groups were divided using the K-mean technique, as they showed different groups of MPIs.

Also, the figures make it possible to identify that there was an improvement in poverty indicators (Figures 1a and 1b). This change is especially visible for the states of Mato Grosso and Rondônia. Most of the observed improvement is in the urban environment. In a specific analysis of the municipalities, it is possible to verify that only the population living in the urban areas of 42 municipalities showed a worsening (increase) in the indicator from 2000 to 2010; in turn, in the rural environment, 177 municipalities showed a worsening in the MPI over the study period.

The average decrease in poverty intensity in the 10-year period was 1.29% but 252 municipalities showed worsening levels of deprivation. As can be seen in Figure 2, a large part of this drop in the intensity of poverty occurred in the municipalities of the State of Amazonas and in the Mesoregion of Marajó in Pará. In Figures 2a and 2b, it is noteworthy how the reduction in intensity is more associated with greater urban concentration, while it can be seen in Figures 3a and 3b that the increase in the incidence indicator, i.e. the worsening of deprivation, occurs most prominently in the rural sector of the Legal Amazon.

### Aspects of health and sanitation conditions in the composition of the MPI

To better understand how the field of health can support the analysis of the MPI, two important indicators were observed: the contribution of health in the composition of the MPI and the contribution of sanitation.

Based on the descriptive statistics for 756 of the 772 municipalities in the Brazilian Legal Amazon, Table 1 evinces two noteworthy aspects: the first is that, from one decade to the other, on average, the health dimension increased its contribution to the MPI determination, that is, compared to the other dimensions, the health condition of the municipalities worsened from 2000 to 2010. On average, 13% of the MPI was derived from health conditions in 2010. By 2015, 15% of the health dimension became responsible for the composition of the MPI.

**Table 1:** Descriptive results of the multidimensional poverty index (MPI) indicators from 2000 and 2010 (number of households, mean, standard deviation [SD], minimum and maximum values) (n = 756).

Variables	Mean	SD	Minimun	Maximun
Intensity (A) 2000	0.35	0.01	0.31	0.39
Intensity (A) 2010	0.35	0.02	0.29	0.45
Incidence (H) 2000	0.58	0.16	0.12	0.92
Incidence (H) 2010	0.40	0.20	0.05	0.91
MPI 2000	0.21	0.06	0.04	0.35
MPI 2010	0.14	0.08	0.02	0.40
Health dimension 2000 (%)	13.00	4.00	0.00	25.00
Health dimension 2010 (%)	15.00	5.00	2.00	30.00
Sanitation conditions 2000 (%)	14.00	2.00	7.00	18.00
Sanitation conditions 2010 (%)	7.00	2.00	1.00	11.00

Source: prepared by the authors based on the 2000 and 2010 Brazilian Demographic Censuses ^39^ ^,^ ^40^.

Over the years, there has been significant improvement in Sanitation Conditions in the Legal Amazon (the sums of the contributions of the indicators for lack of sewage, lack of garbage collection, and lack of piped water were used to compose the sanitation conditions). The proportion of people living in precarious conditions decreased from 14% in 2000 to 7% in 2010.

Regarding health conditions, the State of Mato Grosso raises the most concern in the composition of poverty, as shown in Table 2. Of the 126 municipalities analyzed, only nine presented a decrease in the health component of multidimensional poverty in the Amazon, whereas the remaining 117 experienced an increase (worsening) in the health indicator (infant mortality) in the composition of poverty.

**Table 2:** Number of municipalities that worsened and improved the health dimension in the composition of the multidimensional poverty index (MPI) from 2000 to 2010 by Federative Unit (UF, acronym in Portuguese). Legal Amazon, Brazil.

UF	Variation in health condition		Total	
	Worsened		Improved		
	n	%	n	%	n
Acre	14	64	8	36	22
Amazonas	28	45	34	55	62
Amapá	12	75	4	25	16
Maranhão	92	51	89	49	181
Mato Grosso	117	93	9	7	126
Pará	81	57	62	43	143
Rondônia	46	88	6	12	52
Roraima	4	27	11	73	15
Tocantins	112	81	27	19	139
Total	506		250		756

Source: prepared by the authors based on the 2000 and 2010 Brazilian Demographic Censuses ^39^ ^,^ ^40^.

In total, only 250 out of the 756 municipalities analyzed experienced a decrease in the health dimension of the MPI. Tocantins held the second worst situation, with 112 out of 139 municipalities showing a worsening in the health contribution to the MPI, followed by Maranhão (92) and Pará (81). The states of Amazonas and Roraima were the only ones that showed more municipalities with a decrease than an increase in contribution.

**Figure 4:**
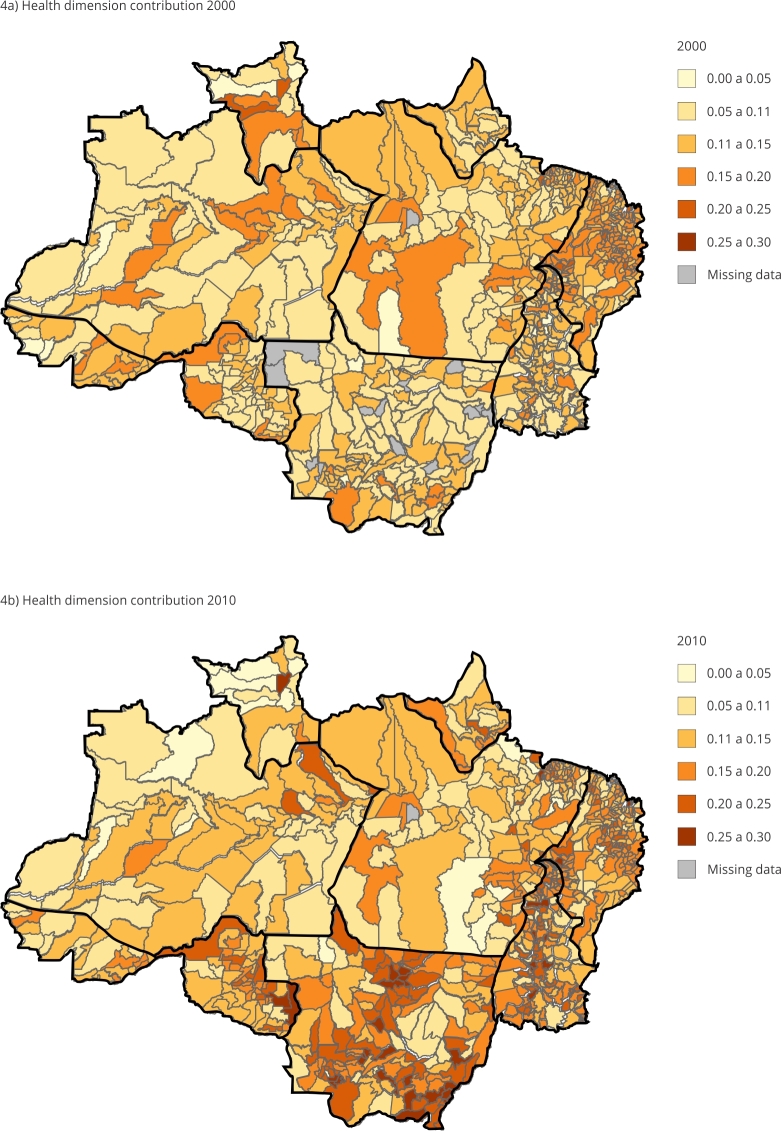
Spatial evolution of the health dimension contribution to multidimensional poverty index (MPI) from 2000 to 2010. Legal Amazon, Brazil.

Source: prepared by the authors based on the 2000 and 2010 Brazilian Demographic Censuses ^39^ ^,^ ^40^.

Figures 4a and 4b show how the contribution of the health dimension to the MPI is spatially distributed on a percentage scale.

**Figure 5:**
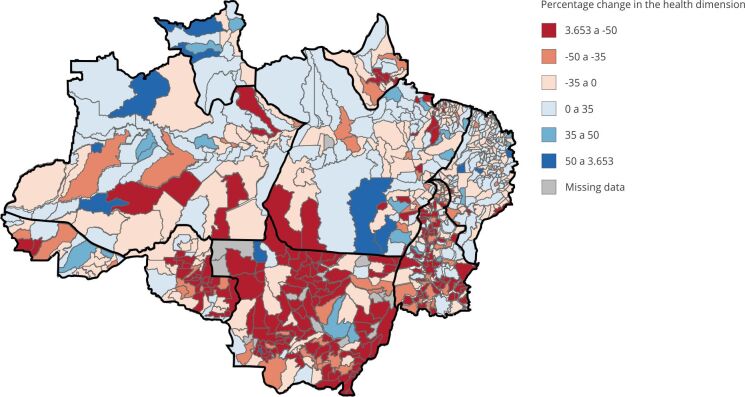
Percentage change in the health dimension of the multidimensional poverty index (MPI) from 2000 to 2010. Legal Amazon, Brazil.

Source: prepared by the authors based on the 2000 and 2010 Brazilian Demgraphic Censuses ^39^ ^,^ ^40^.

Figure 5 highlights how the worsening of health is spatially distributed as a determinant in the composition of the MPI. The municipalities in red indicate those that have worsened and the percentage variation of this worsening, while those that have improved are shown in blue.

What can be highlighted in the results is the number of municipalities that showed more than 35% worsening in the health dimension in the composition of the MPI, most of which are located in the State of Mato Grosso.

Note that the use of the MPI - Brazilian Legal Amazon paves the way for future and more detailed analyses, including the identification of spatial patterns not explored in this study. Additionally, this indicator enables investigate correlations between variables and the cause-and-effect relation between variables associated with the index and specific public policies, both in the field of public health and in other areas. In other words, MPI not only provides a comprehensive overview of poverty in the region, but also offers valuable insights into how policies can be targeted to address the various dimensions of poverty more effectively.

## Discussion

As highlighted by Santos et al. ^26^, it is clear that the current databases available for the proper application of the Alkire-Foster methodology are still unable to satisfactorily cover significant elements of rural settings. Specifically in the Amazon Region, the questionnaire does not include answers that allow for a more precise analysis of living and consumption patterns in households linked to rural production systems operated by family farmers, considering their microeconomic logics ^2^ ^,^ ^41^ ^,^ ^42^. Thus, when creating economic and social indicators, aspects such as productivity, consumption, and purchasing power (all assessed in the short term) are generally considered to be sufficient parameters for assessing the well-being of populations. These parameters, when associated with the category of poverty, are linked to the processes that generate economic and social development. These indicators, however, often simplify the complexity of the situation into a single measure.

We also highlight that discussions about environmental indicators in Brazil’s Legal Amazon generally focus on deforestation and wildfires, disregarding other indicators that could guide policies and decisions for the development of the region. As for health indicators, they often focus on diseases without integrating health issues with socioeconomic sustainability and the well-being of populations ^9^. The outcomes of the analysis provide parameters for the relationship between health and other dimensions in the Legal Amazon, using the MPI as a metric. From 2000 to 2010, there was an increase in the contribution of the health dimension to the composition of the MPI in the region, indicating a possible worsening of the health conditions of the municipalities during this decade.

In contrast, there was a significant improvement in sanitation conditions, suggesting that the sanitation policies implemented in the region were effective during the period. The results corroborate Vicentin & Minayo ^1^ who, despite showing improvements, have highlighted that seeking solutions to these problems can create economic and innovation opportunities, either by applying local solutions more efficiently or by adopting alternative solutions that are more compatible with the resources and limitations of the local economy.

When analyzing the composition of multidimensional poverty in the health indicator by state, Mato Grosso stood out as an area of concern. Most municipalities in this state experienced an increase in the contribution of health to the MPI, indicating a possible worsening of health conditions (infant mortality). Moreover, other states such as Tocantins, Maranhão, and Pará also showed a significant number of municipalities in which health held a greater contribution to the MPI, suggesting similar challenges in relation to health in these regions during the period analyzed.

In summary, the results highlight the complex interaction between health, education, living standards, work and assets, and poverty in the Legal Amazon, identifying areas of success and persistent challenges that require continued attention and targeted public policies to improve living conditions and reduce multidimensional poverty. The specific analysis of each dimension and its relations with the environmental and socioeconomic complexity of the region bring a rich field of possibilities, leading to perspectives that go beyond the criterion of income for the identification of vulnerability and poverty.

Therefore, when debating development alternatives and formulating policies for regional economies in the Brazilian Amazon, it is crucial to use methodologies for constructing poverty indicators and assessing their impact. When we consider poverty as a substantial parameter to assess the degree of freedom necessary to achieve human development ^10^, ideally, we should create multiple indicators capable of evaluating the continuous and multidimensional process of improving the conditions of territorial development of populations, integrating them into the production of their livelihoods.

An alternative has been the use of a group of indicators that considers the multidimensional aspects of poverty and development, going beyond the components related only to people’s consumption or income. In this group, the MPI, based on the capabilities approach and the Alkire-Foster decomposition method ^15^ ^,^ ^17^ ^,^ ^24^, stands out for two important reasons. First, its association with the United Nations Development Programme (UNDP) strengthens its presence as an indicator in global development-related agendas such as the Sustainable Development Goals 2030 Agenda and the UN-Habitat New Urban Agenda ^43^. Second, its methodological approach, adopting an axiomatic basis for the construction of the measure, is flexible enough to accommodate various dimensions and variables, opening space for debate about which dimensions and variables should be included in the information systems that feed national statistics.

It is also important to highlight the need to assess the complexity of socioeconomic indicators for rural areas. According to a partnership between the Food and Agriculture Organization of the United Nations (FAO) and the Oxford Poverty and Human Development Initiative (OPHI) ^44^, the well-being variables associated with rural areas differ from those associated with the urban areas.

Thus, Alkire-Foster method ^24^ and multidimensional indicators can bring new perspectives as tools to assess the well-being of populations in Brazil’s Legal Amazon, despite data limitations. The results of the MPI presented in this study reveal crucial opportunities and challenges that we need to overcome when considering MPI as a tool to discuss poverty and development in the Brazilian Amazon.

## Conclusions

Despite their limitations, multidimensional indicators reveal opportunities for debating health policies in the Brazilian Amazon. The use of the MPI as a socioeconomic indicator would allow researchers to look beyond income. As aforementioned, variables that capture poverty and socioeconomic vulnerabilities are directly linked to the spread of disease, mortality rates, availability of public health infrastructure, and the types of treatment that are available to the population, especially when we consider the rural space of the Amazon and its protagonists. From 2000 to 2010, the direct association between rural Amazonia and the persistence of poverty was, to say the least, questionable. The incorporation of new variables to measure rural deprivations, following the implementation of programs and policies that considered the economies of rural technological systems associated with agroforestry systems managed by small farmers, revealed an improvement in the incidence (H) and intensity (A) components of multidimensional poverty in the Eastern Amazon, where these systems still predominate.

For a development agenda in the Amazon, it is essential that economies related to the biome are considered as strategies for regional development that is socially just and environmentally responsible. It is imperative to seek better indicators of poverty, especially in the rural context, so that we can make informed decisions that consider both the indicators and the forest and the people who depend on it.
