# Supplementary material for: Multidimensional poverty indicators in Brazil’s Legal Amazon
Source: Cad Saude Publica. 2025 Mar 24;41(Suppl 1):e00212223. doi: 10.1590/0102-311XEN212223 (PMC11940851; doi:10.1590/0102-311XEN212223)
Supplement: Translation

# Translation: Indicadores de pobreza multidimensionais na Amazônia Legal

**DOI:** 10.1590/0102-311XPT212223

**Authors:** Camila de Moura Vogt, Ricardo Bruno Nascimento dos Santos, Danilo Araújo Fernandes

**Affiliations:** 1 Universidade Federal de Santa Maria, Santa Maria, Brasil.; 2 Universidade Federal do Pará, Belém, Brasil.

**Keywords:** Ecossistema Amazônico, Pobreza, Indicadores Sociais, Renda

## Abstract

Na pesquisa em saúde pública, os indicadores socioeconômicos desempenham um papel crucial, sendo essenciais para entender a relação entre pobreza e questões de saúde, bem como para direcionar a alocação de recursos na infraestrutura de saúde pública e na oferta de tratamentos adequados à população. Tradicionalmente, a renda tem sido o principal indicador usado para medir a pobreza, estabelecendo uma linha que considera quem está acima dela como não pobre ou não vulnerável, e quem está abaixo como pobre ou vulnerável. Essa análise unidimensional da condição, entretanto, tem suas limitações, particularmente em regiões com complexidade epidemiológica elevada e populações altamente vulneráveis como a Amazônia Legal. Esse trabalho discute e calcula uma abordagem multidimensional da pobreza na Amazônia Legal a partir do índice de pobreza multidimensional (IPM), conforme a metodologia Alkire-Foster (2010). Como indicador socioeconômico, o IPM considera não apenas a renda, mas também outros âmbitos relacionados à saúde pública que afetam o bem-estar das populações. A utilização de um indicador multidimensional permite uma compreensão mais completa das condições socioeconômicas e de vida, facilitando a formulação de políticas públicas. Os resultados do indicador calculado, IPM - Amazônia Legal, mostram diferenças importantes nas características da pobreza rural, bem como na intensidade e incidência dessa pobreza, que não são identificadas pelos indicadores de renda unidimensionais. O trabalho conclui que a abordagem multidimensional é fundamental para lidar com as complexas questões de saúde pública e desenvolvimento socioeconômico na região.

## Introdução

Indicadores socioeconômicos são de extrema importância para estudos na saúde pública, em especial em áreas com alta complexidade epidemiológica e grande vulnerabilidade das populações como é o caso da Amazônia Legal ^1^ ^,^ ^2^ ^,^ ^3^ ^,^ ^4^. Variáveis que consigam mensurar como a pobreza e as vulnerabilidades socioeconômicas se relacionam diretamente com proliferação de doenças e mortalidade indicam a infraestrutura de saúde pública e os tipos de tratamentos que serão ofertados para a população. A caracterização da pobreza e vulnerabilidades, entretanto, é um tema controverso para o debate de políticas públicas. O que define uma pessoa pobre ou socioeconomicamente vulnerável não é algo estático, podendo variar no tempo e na cultura. Tradicionalmente, a pobreza é medida pela renda e ligada com a capacidade de consumo dos indivíduos.

Porém, a distinção entre pobreza e vulnerabilidade é fundamental para compreender as complexidades das condições socioeconômicas e as necessidades das populações em situação de privação. Enquanto a pobreza se refere à escassez de recursos materiais básicos, como renda, alimentação e moradia, a vulnerabilidade abrange a exposição a riscos e a capacidade de enfrentá-los ou se recuperar deles. Embora a pobreza possa ser um fator contribuinte para a vulnerabilidade, a última é influenciada por uma variedade de aspectos como exclusão social, discriminação, falta de acesso a serviços essenciais e eventos adversos como desastres naturais e conflitos armados. Compreender essa distinção permite desenvolver políticas e intervenções mais eficazes para atender às necessidades específicas das comunidades vulneráveis ^5^ ^,^ ^6^ ^,^ ^7^ ^,^ ^8^ ^,^ ^9^ ^,^ ^10^.

O método mais comum de mensuração é a criação de uma linha de pobreza, ou seja, um valor monetário de referência que compreenda um limite em que quem está acima é considerado não-pobre (ou não vulnerável) e quem está abaixo pobre (ou vulnerável). Essa escolha metodológica é normalmente justificada pela conveniência de dados ou pela capacidade que a limitação monetária tem de definir o bem-estar de uma população. Afinal, a partir da limitação em adquirir determinada cesta de bens, é possível entender muito sobre o nível de vulnerabilidade que a família ou o indivíduo irá lidar ^9^ ^,^ ^11^ ^,^ ^12^ ^,^ ^13^.

Mas a análise unidimensional da situação de pobreza não é consenso para expressar o desenvolvimento humano. Apesar da importância da dimensão renda, sabe-se que existem outros fatores que podem ser associados com a situação de pobreza. Acesso a serviços públicos, saúde, educação, trabalho e segurança estão fortemente ligados ao bem-estar, mas não são totalmente explicados pela capacidade de consumo de uma população. Da mesma forma, existem diferenças entre os níveis de pobreza e os tipos de políticas públicas necessárias para cada população específica, que ficam sub identificadas em métodos unidimensionais de mensuração ^11^.

A Amazônia Legal compreende uma extensa porção da Amazônia, que é a maior floresta tropical do mundo, localizada dentro do território brasileiro. Essa região abrange aproximadamente 58,93% do território do Brasil, com uma área total de 5.015.146,008km^2^. Sua definição se baseia em critérios legais e administrativos, englobando nove estados brasileiros: Acre, Amapá, Amazonas, Maranhão, Mato Grosso, Pará, Rondônia, Roraima e Tocantins, abrangendo um total de 772 municípios ^14^. Ao longo da história, a Amazônia tem sido frequentemente percebida de forma dualista. Por um lado, é vista como marcada pela pobreza, vulnerabilidade e atraso; enquanto por outro é considerada rica devido ao seu potencial de exploração de recursos naturais. Essa abordagem, que separa as dimensões sociais e econômicas da realidade ambiental, tem dificultado a compreensão dos diferentes modos pelos quais as populações locais utilizam a biodiversidade e como isso afeta o bem-estar e as vulnerabilidades delas.

Dadas as complexidades do território da Amazônia Legal, a utilização de indicadores socioeconômicos que contemplem todas as características da região é uma tarefa desafiadora. Nesse aspecto, a escolha de indicadores socioeconômicos pode levar a concepções e avaliações muitas vezes equivocadas. Este estudo tem como propósito examinar os índices multidimensionais utilizados para identificar a pobreza e enfatizar o índice de pobreza multidimensional (IPM) na região da Amazônia Legal, que é um indicador que oferece diversas vantagens metodológicas para a compreensão da situação socioeconômica da região. Para tanto, foi calculado o IPM − Amazônia Legal para o território e exploradas as maneiras pelas quais esse pode transcender a abordagem puramente monetária na avaliação das políticas públicas devido à sua metodologia flexível, que leva em consideração diversas características das famílias e as integra em um único índice. Essa abordagem possibilita uma análise mais abrangente e detalhada das condições de vida das populações locais, proporcionando uma compreensão mais precisa das necessidades e desafios enfrentados pela Região Amazônica.

Considerando essa problemática, abordagens multidimensionais têm ganhado destaque no que concerne à avaliação da pobreza. Diferentemente das abordagens unidimensionais, que tradicionalmente se limitam a medir a pobreza com base em um único indicador, as abordagens multidimensionais consideram diversos aspectos da vida das pessoas, permitindo uma melhor avaliação da influência de seus componentes ligados a especificidade do território e resultando em uma compreensão mais completa das privações que enfrentam. Essas abordagens multidimensionais podem ser classificadas em três categorias principais: as axiomáticas, como proposto por Alkire & Santos ^15^ e de teoria da informação; as metodologias *fuzzy*; e as teorias de variáveis latentes.

Entre as abordagens multidimensionais, os métodos de *dashboard* ^16^ e os índices compostos são amplamente utilizados, especialmente no contexto do desenvolvimento humano, conforme descrito por Alkire & Santos ^(^ ^15^ e Alkire et al. ^17^. Um exemplo notável é o programa da Organização das Nações Unidas para os Objetivos de Desenvolvimento Sustentável (ODS), que utiliza um *dashboard* composto por 49 indicadores para monitorar 17 objetivos e 169 metas ^18^ ^,^ ^19^ ^,^ ^20^. Para o Brasil, esse método foi utilizado em diversos estudos, entretanto sem conseguir manter o rigor temporal com relação às bases de dados disponíveis e comparações entre territórios, especialmente entre áreas metropolitanas (com maior disponibilidade de microdados) e não metropolitanas (com microdados limitados) ^19^.

O índice de desenvolvimento humano (IDH) ^(^ ^21^ e o índice de pobreza humana (IPH) ^22^ também são exemplos de índices compostos que incorporam múltiplas dimensões relacionadas à pobreza. No entanto, apesar de sua ampla utilização, possuem limitações na identificação de privações simultâneas e na caracterização das heterogeneidades espaciais, especialmente as diferenças entre áreas rurais e urbanas.

Outros indicadores multidimensionais que avaliam de forma mais ampla as vulnerabilidades sociais também são utilizados para a Região Amazônica. O índice de progresso social (IPS) é uma medida abrangente, que avalia o desempenho social e ambiental das nações sem depender apenas do desenvolvimento econômico. Criado em 2013 pela Social Progress Imperative (Imperativo de Progresso Social) ^23^, o IPS reconhece que o crescimento econômico por si só não garante o progresso social, podendo levar a problemas como exclusão, descontentamento social e degradação ambiental. Em sua estrutura de 2018, incluiu 51 indicadores sociais e ambientais distribuídos em três dimensões (Necessidades humanas básicas, Fundamentos para o bem-estar e Oportunidades) e 12 componentes, baseando-se em dados públicos disponíveis *online*.

Apesar de amplamente utilizadas e possuírem facilidade de mensuração em razão de trabalharem com números de agregados nacionais, estaduais e municipais, as metodologias multidimensionais como ODS, IDH, IPH e IPS falham em identificar privações simultâneas e caracterizar de maneira adequada as heterogeneidades do espaço, em especial as diferenças rurais e urbanas. Assim, não são as metodologias mais apropriadas para a mensuração das vulnerabilidades específicas dos domicílios brasileiros. Já as medidas axiomáticas, como o IPM, apresentam várias características convenientes. Primeiro, cumprem os dois passos necessários para a medição da pobreza: identificar os pobres e agregar as informações em um único indicador. Em segundo lugar, seus portfólios incluem medidas que só se aplicam quando os indicadores são cardinais, mas também incluem as que se aplicam quando os indicadores são ordinais ^17^ ^,^ ^24^. Adicionalmente, o IPM, apesar de ser um indicador sintético, permite a análise de cada uma das dimensões que caracterizam o entendimento de pobreza e vulnerabilidade de modo separado, sendo possível entender o peso de cada uma delas na definição da pobreza.

O cálculo do IPM exige a identificação das unidades de análise (normalmente agregados familiares) e a avaliação das privações que enfrentam em várias dimensões. As unidades são consideradas multidimensionalmente pobres quando sua pontuação de privação ultrapassa um limite interdimensional. O IPM Global, baseado no método Alkire-Foster, é utilizado em escala mundial e incorpora cinco dimensões de bem-estar: pobreza monetária; educação; acesso à infraestrutura básica; saúde e nutrição; e segurança ^25^.

No cenário brasileiro, diversos estudos acadêmicos têm aplicado o IPM e outras abordagens multidimensionais para avaliar as condições de vida e bem-estar da população. Eles se baseiam em microdados de pesquisas nacionais, como os Censos Demográficos e a *Pesquisa Nacional por Amostra de Domicílios* (PNAD). A pesquisa acadêmica no Brasil tem abordado uma variedade de dimensões, incluindo saúde, educação, padrão de vida, segurança e acesso a serviços básicos, tanto em áreas urbanas quanto rurais. Esses estudos desempenham um papel fundamental na análise das condições de vida da população brasileira e na identificação de desigualdades regionais, fornecendo informações valiosas para o desenvolvimento de políticas públicas destinadas a melhorar a qualidade de vida em todo o país. Portanto, abordagens multidimensionais têm se mostrado instrumentos importantes para se compreender a complexidade da pobreza e para orientar ações governamentais que visam a promoção do bem-estar e o combate à desigualdade.

Conforme esquematizado por Santos et al. ^26^, no âmbito da pesquisa acadêmica brasileira, diversos estudos abordam a avaliação da qualidade de vida e do bem-estar em diferentes regiões do país, empregando uma variedade de dimensões e indicadores. Bagolin & Ávila ^27^ investigam, em escopo nacional, dimensões relacionadas à saúde, segurança, educação e segurança alimentar, utilizando indicadores como leitos hospitalares, oferta de médicos, mortalidade infantil, expectativa de vida, homicídios, oferta de policiais, analfabetismo, escolaridade e proporção de pessoas com restrição alimentar. Barros et al. ^28^ se concentram em dimensões de vulnerabilidade, acesso ao conhecimento, acesso ao trabalho, escassez de recursos, desenvolvimento infantil e carências habitacionais, comparando áreas rurais e urbanas em nível nacional. Seus indicadores incluem fecundidade, atenção e cuidados especiais com diferentes grupos etários, dependência demográfica e presença da mãe, entre outros.

Diversos outros estudos recentes têm conseguido resultados importantes no sentido de demonstrar a complexidade dos impactos de diversas dimensões e especificidades da pobreza e vulnerabilidade em diferentes regiões do país ^29^ ^,^ ^30^ ^,^ ^31^ ^,^ ^32^ ^,^ ^33^ ^,^ ^34^ ^,^ ^35^ ^,^ ^36^.

Para a Amazônia Legal, a escolha de indicadores que abordem a pobreza e características socioeconômicas de modo multidimensional se torna ainda mais pertinente. As desigualdades econômicas intrarregionais do local são significativas: enquanto algumas áreas podem se beneficiar (direta ou indiretamente) da melhoria de infraestrutura e geração de empregos em determinados setores econômicos considerados mais dinâmicos (como a mineração e o desenvolvimento da agropecuária), outras sofrem com a falta completa de infraestrutura e acesso a serviços básicos. Essas disparidades se manifestam no acesso desigual a serviços de saúde, educação, emprego e segurança, por exemplo.

Por outro lado, muitas regiões que se beneficiam do dinamismo de setores econômicos sofrem com as transformações aceleradas nas condições de acesso a recursos naturais considerados de fundamental importância para a garantia de segurança alimentar e manutenção dos meios de vida das populações tradicionais, que sobrevivem do acesso a áreas comuns, rios, lagos e recursos disponíveis nas florestas. Adicionalmente, muitas áreas da Amazônia Legal carecem de infraestrutura básica como estradas e eletricidade, o que dificulta o acesso a serviços e oportunidades econômicas. O isolamento resultante cria um ciclo de desvantagem, impedindo o alcance a mercados e oportunidades de emprego.

As estratégias de desenvolvimento e redução das vulnerabilidades para a população amazônica combinam uma série de iniciativas que divergem fortemente com relação aos modelos e indicadores socioeconômicos. O arco do desmatamento, como é conhecida a região que abrange partes dos estados do Maranhão, Tocantins, Pará, Mato Grosso, Rondônia e Acre é considerada uma das fronteiras ativas de uso da terra em termos de desmatamento e queimadas, mas também é uma região com altos indicadores de PIB *per capita* e IDH ^37^. A controvérsia entre crescimento econômico e desmatamento é recorrente no debate sobre um modelo de crescimento e desenvolvimento em toda a Região Amazônica.

Conforme Guedes et al. ^38^ em pesquisa realizada na região de Altamira, no Pará, as condições multidimensionais de bem-estar revelam que algumas estratégias de uso da terra economicamente viáveis por pequenos proprietários (por exemplo, pastagens) podem ter importantes implicações ecológicas para a paisagem regional, o que afeta a garantia e o direito de acesso a bens e serviços que são de usufruto e benefício coletivo para um grande contingente populacional na região. Em outros estudos da Amazônia Legal que usam indicadores multidimensionais, é clara a relação complexa entre questões estruturais da região e fatores que vão além dos indicativos de renda *per capita* ^4^ ^,^ ^26^ ^,^ ^36^ ^,^ ^38^.

## Metodologia

A metodologia do IPM de Alkire-Foster ^24^ consegue ser segmentada em duas fases: a identificação e a agregação. De maneira intuitiva, elas podem ser separadas em etapas, sendo consideradas seis na fase de identificação. A primeira etapa consiste em definir o conjunto de indicadores que serão considerados na medida multidimensional, levando em conta que os dados para eles precisam estar disponíveis para a mesma pessoa ou família observada. Na segunda e terceira etapa são trabalhados os pontos de corte de cada indicador e a definição das carências. Finalmente, nas últimas etapas é selecionado o peso ou o valor relativo que cada indicador possui, de modo que somem um. É então criada a “pontuação de privação” e determinado normativamente o limite da pobreza, ou seja, a proporção de privações ponderadas que uma pessoa precisa experimentar para ser considerada multidimensionalmente pobre.

Já na fase de agregação para a criação do indicador, são consideradas sete etapas adicionais. Primeiro, realiza-se o cálculo da proporção de pessoas que foram identificadas como multidimensionalmente pobres na população. Esse é o índice de pobreza multidimensional *H*, também chamado de incidência de pobreza multidimensional. Em seguida, é calculada a intensidade da pobreza multidimensional *A*, ou amplitude da pobreza, que é a proporção média de privações. São ainda calculados os índices *M*_*0*_ , *M*_*1*_ e *M*_*2*_ considerando somente as populações identificadas como pobres e sendo *M*_*0*_ o índice ajustado (que normalmente é utilizado como indicador, sendo *M*_*0*_ = H * A) ou a soma das privações ponderadas que os pobres experimentam divididos pela população total. Já *M*_*1*_ leva em consideração a diferença média de pobreza nos casos em que as pessoas pobres são privadas, ou a profundidade da pobreza *G*, sendo *M*_*1*_ = H * A * G, ou a soma das diferenças de privação, dividida pela população total. Finalmente, o indicador *M*_*2*_ vai levar em consideração a severidade da pobreza (*S*), que é a média das diferenças de privação ao quadrado. Assim, *M*_*2*_ = H * A * S, ou a soma das lacunas de privação ao quadrado, dividida pela população total.

O índice reflete o número de privações que o indivíduo sofre em um determinado momento. Assim, os considerados pobres não necessariamente apresentam todas, mas um conjunto de *i* privações básicas *c*_*i*_ sobrepostas. A variável *k* é o ponto de corte do indicador de pobreza e reflete a soma ponderada, na qual o indivíduo é considerado multidimensionalmente pobre. Assim, o IPM é a multiplicação de dois termos: o *H*, incidência da pobreza, $H=\frac{q}{n}$ , sendo *q* o número de indivíduos multidimensionalmente pobres e *n* a população total; e o *A*, a intensidade da pobreza (Equação 1) ^24^.

$$A=\frac{1}{q}\sum_{i=1}^{q}c_{i}\left(k\right)$$ (Equação 1)

Dessa forma, também podemos reescrever o indicador IPM conforme Equação 2:

$$IPM=\frac{1}{n}\sum_{i=1}^{n}c_{i}\left(k\right)$$ (Equação 2)

A contagem máxima de carências é 100%, e no IPM - Amazônia Legal foram consideradas quatro dimensões, em que cada uma recebeu a ponderação de 1/4. Assim, a contagem máxima em cada dimensão ou o ponto de corte da pobreza é de 25%. Cada indicador foi ponderado, dividindo a ponderação (1/4) pelo número de indicadores. O ponto de corte no IPM - Amazônia Legal foi definido de maneira proporcional ao número de dimensões. Para identificar os domicílios multidimensionalmente pobres, soma-se as carências de cada dimensão para obter posteriormente a carência total correspondente de cada casa.

A base de dados é proveniente dos microdados domiciliares dos Censos Demográficos de 2000 e 2010 ^39^ ^,^ ^40^. Eles contemplam todos os municípios da Amazônia Legal, englobando nove estados brasileiros e um total de 756 dos 772 municípios da região ^39^ ^,^ ^40^ (os que foram criados entre 2000 e 2010 e os sem informações entre os dois anos foram excluídos da análise). Todas as informações foram geradas conforme metodologia Alkire-Foster para o cálculo do IPM municipal da Amazônia Legal.

O Quadro 1 destaca as variáveis utilizadas e a dimensão a que cada uma delas pertencem.

Dessa forma, o IPM - Amazônia Legal foi composto por 16 indicadores para a situação rural e urbana em quatro diferentes dimensões. O conjunto de indicadores foi selecionado a partir de estudos recentes ^4^, conforme as informações coletadas no questionário da amostra para o referido levantamento censitário.

**Quadro 1:** Descrição das variáveis, dimensões e pesos usados no índice de pobreza multidimensional (IPM).

VARIÁVEL	DIMENSÃO	PESO DO INDICADOR NO IPM
Óbitos de filhos nascidos vivos	Saúde	1/4
Adultos sem Ensino Fundamental	Educação	1/12
Responsável pelo domicílio não sabe ler nem escrever	Educação	1/12
Crianças fora da escola	Educação	1/12
Não possui esgotamento sanitário	Condições de vida	1/28
Lixo sem destino adequado	Condições de vida	1/28
Não possui energia elétrica	Condições de vida	1/28
Sem acesso a água canalizada	Condições de vida	1/28
Mora em domicílio improvisado ou coletivo	Condições de vida	1/28
Densidade do domicílio superior a cinco pessoas	Condições de vida	1/28
Condição do terreno cedido	Condições de vida	1/28
Renda proveniente apenas de programas sociais	Condições de trabalho e bens privados	1/20
Responsável pelo domicílio sem trabalho remunerado	Condições de trabalho e bens privados	1/20
Não possuem geladeira	Condições de trabalho e bens privados	1/20
Indivíduos que não possuem tecnologia digital	Condições de trabalho e bens privados	1/20
Indivíduos que não possuem pelo menos 2 bens privados	Condições de trabalho e bens privados	1/20

Fonte: elaboração própria com base em Censos Demográficos de 2000 e 2010. ^39^ ^,^ ^40^.

## Resultados

Os resultados do IPM - Amazônia Legal podem ser divididos e analisados considerando questões específicas, como predominância urbana/rural e questões territoriais, ou segregações específicas, considerando domicílios, regiões censitárias, municípios, estados e finalmente a Região da Amazônia Legal como um todo. Entretanto, os resultados apresentados se limitam a analisar a evolução das diferentes regiões para os municípios e estados ao longo dos anos.

As Figuras 1, 2 e 3 mostram o IPM e os dois componentes que o constituem, incidência (H) e intensidade (A), entre os anos de 2000 e 2010 para as áreas predominantemente urbanas e rurais, conforme classificação do Instituto Brasileiro de Geografia e Estatística (IBGE) dos municípios da Amazônia Legal. Verificando seu perfil de variação entre os anos, os grupos de divisão foram constituídos pela técnica de K-médias para o período, visto que possuíam grupos diferentes de IPMs.

**Figura 1:** Evolução espacial do índice de pobreza multidimensional (IPM) entre os anos de 2000 e 2010. Amazônia Leal, Brasil.

Fonte: elaboração própria com base nos Censos Demográficos de 2000 e 2010 ^39^ ^,^ ^40^.

**Figura 2:** Evolução espacial da intensidade da pobreza multidimensional (A) entre os anos de 2000 e 2010. Amazônia Leal, Brasil.

Fonte: elaboração própria com base em Censos Demográficos de 2000 e 2010 ^39^ ^,^ ^40^.

**Figura 3:** Evolução espacial da incidência de pobreza multidimensional (H) entre os anos de 2000 e 2010. Amazônia Leal, Brasil.

Fonte: elaboração própria com base em Censos Demográficos de 2000 e 2010 ^39^ ^,^ ^40^.

É possível identificar nas figuras que houve uma melhora dos indicadores de pobreza (Figuras 1a e 1b), com essa mudança sendo visível principalmente nos estados do Mato Grosso e Rondônia. A maior parte da melhoria observada está no ambiente urbano. Em análise específica nos municípios, é possível constatar que apenas a população residente no ambiente urbano de 42 municípios teve piora (aumento) no indicador de 2000 a 2010 e, por sua vez, 177 municípios no ambiente rural tiveram piora no IPM entre o decênio observado.

A queda média da intensidade da pobreza no período de 10 anos foi de 1,29%, porém 252 municípios tiveram piora em seus níveis de carência. Como pode ser observado na Figura 2, grande parte dessa queda na intensidade da pobreza ocorreu nos municípios do Estado do Amazonas e na Mesorregião do Marajó, no Pará. Nas Figuras 2a e 2b, é notável como a redução na intensidade está mais associada à parte com maior concentração urbana, enquanto se observa nas Figuras 3a e 3b que o aumento do indicador de incidência, ou seja piora das carências, ocorre com maior destaque no setor rural da Amazônia Legal.

### Aspectos da saúde e condições de saneamento na composição do IPM

Para auxiliar em como o campo da saúde pode ajudar na análise do IPM, foram observados dois importantes indicadores: a contribuição da saúde na composição do IPM; e a contribuição das condições de saneamento.

Como pode ser observado na Tabela 1, a partir das estatísticas descritivas para 756 dos 772 municípios da Amazônia Legal, dois aspectos são importantes. O primeiro é de que, de um decênio para o outro, em média a dimensão saúde aumentou sua contribuição na determinação do IPM. Ou seja, diante das demais dimensões, a condição de saúde dos municípios pioraram de 2000 a 2010. Em média 13% do IPM era proveniente dela, e em 2010, 15% da dimensão saúde passou a ser responsável pela composição do índice.

**Tabela 1:** Resultados descritivos dos Indicadores do índice de pobreza multidimensional (IPM) entre 2000 e 2010 (número de domicílios, média, desvio padrão [DP], valores mínimos e máximos) (n = 756).

Variáveis	Média	DP	Mínimo	Máximo
Intensidade (A) 2000	0,35	0,01	0,31	0,39
Intensidade (A) 2010	0,35	0,02	0,29	0,45
Incidência (H) 2000	0,58	0,16	0,12	0,92
Incidência (H) 2010	0,40	0,20	0,05	0,91
IPM 2000	0,21	0,06	0,04	0,35
IPM 2010	0,14	0,08	0,02	0,40
Dimensão saúde 2000 (%)	13,00	4,00	0,00	25,00
Dimensão saúde 2010 (%)	15,00	5,00	2,00	30,00
Condições de saneamento 2000 (%)	14,00	2,00	7,00	18,00
Condições de saneamento 2010 (%)	7,00	2,00	1,00	11,00

Fonte: elaboração própria com base em Censos Demográficos de 2000 e 2010 ^39^ ^,^ ^40^.

Ao longo dos anos, houve uma significativa melhoria nas condições de saneamento na Amazônia Legal (foram utilizadas as somas das contribuições dos indicadores de Não possui esgotamento sanitário, não possui coleta de lixo e ausência de água encanada para a composição das condições de saneamento). A proporção de pessoas vivendo em condições precárias diminuiu de 14% em 2000 para 7% em 2010.

No que se refere à condição de saúde, o estado que mais causa preocupação na composição da pobreza é o Mato Grosso. Conforme a Tabela 2, apenas 9 dos 126 municípios analisados tiveram queda na composição da saúde na pobreza multidimensional da Amazônia; os 117 restantes tiveram aumento (piora) no indicador de saúde (mortalidade infantil) na composição da pobreza.

No total, apenas 250 dos 756 municípios analisados tiveram queda na dimensão saúde na composição do IPM. Tocantins foi a segunda pior situação, com 112 dos seus 139 municípios piorando na participação da Saúde no IPM, seguido do Maranhão (92) e do Pará (81). Os estados do Amazonas e Roraima foram os únicos que tiveram mais municípios com queda que com aumento na participação.

**Tabela 2:** Quantitativo de municípios que pioraram e melhoraram a dimensão saúde na composição do índice de pobreza multidimensional (IPM) entre 2000 e 2010, por Unidade da Federação (UF), da Amazônia Legal, Brasil.

UF	Variação na condição de saúde		Total (n)		
	Pioraram		Melhoraram			
	n	%	n	%	
Acre	14	64	8	36	22
Amazonas	28	45	34	55	62
Amapá	12	75	4	25	16
Maranhão	92	51	89	49	181
Mato Grosso	117	93	9	7	126
Pará	81	57	62	43	143
Rondônia	46	88	6	12	52
Roraima	4	27	11	73	15
Tocantins	112	81	27	19	139
Total	506		250		756

Fonte: elaboração própria com base em Censos Demográficos de 2000 e 2010 ^39^ ^,^ ^40^.

As Figuras 4a e 4b mostra como estão distribuídas espacialmente em escala percentual a contribuição da dimensão saúde no IPM.

**Figura 4:** Evolução espacial da contribuição da dimensão saúde no índice de pobreza multidimensional (IPM) entre os anos de 2000 e 2010. Amazônia Legal, Brasil.

Fonte: elaboração própria com base em Censos Demográficos de 2000 e 2010 ^39^ ^,^ ^40^.

A Figura 5 deixa mais evidente como está distribuída espacialmente a piora da saúde como determinante na composição do IPM. Os municípios em graduação vermelha mostram os que tiveram uma piora e a variação percentual dela, e os que melhoraram estão em variação azul.

**Figura 5:** Variação percentual da dimensão saúde no índice de pobreza multidimensional (IPM) entre os anos de 2000 e 2010. Amazônia Legal, Brasil.

Fonte: elaboração própria com base em Censos Demográficos de 2000 e 2010 ^39^ ^,^ ^40^.

O que pode ser ressaltado nos resultados é a quantidade de municípios que tiveram mais de 35% de piora na dimensão saúde na composição do IPM, em sua maior parte localizados no Estado do Mato Grosso.

Salienta-se ainda que a utilização do IPM - Amazônia Legal abre possibilidades para se realizar análises mais detalhadas, incluindo a identificação de padrões espaciais que não foram explorados neste estudo. Além disso, esse indicador possibilita investigar correlações entre variáveis e a relação de causa e efeito entre o índice e políticas públicas específicas, tanto no campo da saúde pública quanto em outras áreas. Isso significa que o IPM não apenas fornece uma visão abrangente da pobreza na região, mas também oferece *insights* valiosos sobre como as políticas podem ser direcionadas para abordar as diversas dimensões da pobreza de forma mais eficaz.

## Discussão

Conforme ressaltado em Santos et al. ^26^, é evidente que as atuais bases de dados disponíveis para a aplicação adequada da metodologia Alkire-Foster ainda não conseguem abranger de maneira satisfatória elementos significativos do ambiente rural, por exemplo. Especificamente na Região Amazônica, o questionário não oferece opções que permitam uma análise mais precisa dos padrões de vida e consumo nos lares vinculados aos sistemas produtivos rurais e operados pelos agricultores familiares, levando em conta suas lógicas microeconômicas ^2^ ^,^ ^41^ ^,^ ^42^. Assim, ao criar indicadores econômicos e sociais, geralmente aspectos como produtividade, consumo e poder de compra (todos avaliados a curto prazo) são considerados parâmetros suficientes para avaliar o bem-estar das populações. Quando relacionados à categoria de pobreza, esses parâmetros estão vinculados aos processos que geram desenvolvimento econômico e social. Entretanto, muitas vezes os indicadores simplificam a complexidade da situação em uma única medida.

Reforça-se ainda que as discussões sobre indicadores ambientais na Amazônia Legal geralmente se concentram no desmatamento e queimadas, sem levar em consideração outros que poderiam direcionar políticas e decisões para o desenvolvimento da região. Quanto aos indicadores de saúde, muitas vezes focam em doenças, sem integrar questões de saúde com a sustentabilidade socioeconômica e o bem-estar das populações ^9^. Os resultados da análise fornecem parâmetros para a relação entre saúde e outras dimensões na Amazônia Legal, utilizando o IPM como métrica. Ao longo do período de 2000 a 2010, observou-se um aumento na contribuição da dimensão da saúde para a composição do IPM na região, indicando uma possível piora nas condições desse âmbito nos municípios durante essa década.

Em contraste, houve uma melhora significativa nas condições de saneamento, sugerindo que as políticas implementadas na região foram eficazes durante o período analisado. Os resultados corroboram com Vicentin & Minayo ^1^ que, apesar de mostrar melhoras, salienta que buscar soluções para esses problemas pode criar oportunidades econômicas e de inovação, seja pela aplicação mais eficiente de soluções locais ou pela adoção de soluções alternativas mais compatíveis com os recursos e limitações da economia local.

Ao analisar a composição da pobreza multidimensional do indicador de saúde por estado, destaca-se o Mato Grosso como uma área de preocupação. A maioria dos seus municípios experimentou um aumento na contribuição da saúde para o IPM, indicando uma possível piora nas condições de saúde (mortalidade infantil). Além disso, outros estados como Tocantins, Maranhão e Pará também apresentaram um número significativo de municípios onde a saúde teve uma contribuição maior para o IPM, sugerindo desafios semelhantes em relação a essa área nessas regiões durante o período analisado.

Em resumo, os resultados destacam a complexa interação entre saúde, educação, condições de vida, trabalho e bens privados, e pobreza na Amazônia Legal, identificando áreas de sucesso e de desafios persistentes que requerem atenção contínua e políticas públicas direcionadas para melhorar as condições de vida e reduzir a pobreza multidimensional. A análise específica de cada uma das dimensões e suas relações com a complexidade ambiental e socioeconômica da região trazem uma rica seara de possibilidades, dando perspectivas que ultrapassam o critério de renda para a identificação de vulnerabilidade e pobreza.

Portanto, é crucial para o debate sobre alternativas de desenvolvimento na Amazônia brasileira que sejam utilizadas essas metodologias para se construir indicadores de pobreza e medir o impacto de medidas ao pensar em políticas para as economias regionais relacionadas ao bioma. Quando consideramos a pobreza como um parâmetro substancial na avaliação do grau de liberdade necessário para se alcançar o desenvolvimento humano ^10^, idealmente deveríamos criar vários indicadores capazes de avaliar o processo contínuo e multidimensional de melhoria das condições de desenvolvimento territorial de suas populações, integrando-as à produção de seus meios de vida.

Uma alternativa tem sido o uso de uma família de indicadores que leva em consideração os aspectos multidimensionais da pobreza e do desenvolvimento, indo além dos componentes relacionados apenas ao consumo ou à renda das pessoas. Nessa família, o IPM, baseado na abordagem das capacitações e no método de decomposição de Alkire-Foster ^15^ ^,^ ^17^ ^,^ ^24^, destaca-se por dois motivos importantes. Primeiro, sua associação com o Programa das Nações Unidas para o Desenvolvimento (PNUD) fortalece sua presença como indicador em agendas globais relacionadas ao desenvolvimento, como a Agenda 2030 dos Objetivos do Desenvolvimento Sustentável (ODS) e a Nova Agenda Urbana da ONU-Habitat ^43^. Em segundo lugar, sua abordagem metodológica, que adota uma base axiomática para a construção da medida, é flexível o suficiente para acomodar diversas dimensões e variáveis, abrindo espaço para discussões sobre quais dimensões e variáveis devem ser incluídas nos sistemas de informação que alimentam as estatísticas nacionais.

Destaca-se ainda a necessidade de avaliação da complexidade dos indicadores socioeconômicos para o meio rural. Conforme parceria entre a Organização das Nações Unidas para a Alimentação e a Agricultura (FAO, acrônimo em Inglês) e a Iniciativa de Pobreza e Desenvolvimento Humano de Oxford (OPHI, acrônimo em inglês) ^44^, as variáveis de bem-estar associadas ao meio rural divergem das associadas ao ambiente urbano.

Assim, a metodologia de Alkire-Foster ^24^ e indicadores multidimensionais podem apresentar novas perspectivas, como ferramentas para se avaliar o bem-estar das populações da Amazônia Legal, apesar das limitações de dados. Os resultados do IPM apresentados neste estudo revelam oportunidades e desafios cruciais que precisamos superar ao considerar esse índice como uma ferramenta para se discutir pobreza e desenvolvimento na Amazônia brasileira.

## Conclusões

Apesar das limitações, os indicadores multidimensionais revelam oportunidades para se debater políticas de saúde na Amazônia Legal. O uso do IPM - Amazônia Legal como um indicador socioeconômico permitiria aos pesquisadores olharem além da renda. Como mencionado anteriormente, variáveis que capturem a pobreza e as vulnerabilidades socioeconômicas estão diretamente ligadas à disseminação de doenças, taxas de mortalidade, disponibilidade de infraestrutura de saúde pública e os tipos de tratamento que serão disponibilizados para a população, principalmente quando levamos em consideração integralmente o espaço rural da Amazônia e seus protagonistas. No período entre 2000 e 2010, a associação direta entre o rural da Amazônia e a persistência da pobreza se mostrou no mínimo questionável. A incorporação de novas variáveis para a medição das privações rurais, após a implementação de programas e políticas que consideraram as economias dos sistemas tecnológicos rurais associados aos sistemas agroflorestais conduzidos por camponeses, revelou evolução nos componentes H e A da pobreza multidimensional na Amazônia oriental, onde esses sistemas ainda predominam.

Para uma agenda de desenvolvimento na Amazônia, é essencial que as economias relacionadas ao bioma sejam consideradas como estratégias para um desenvolvimento regional que seja socialmente justo e ambientalmente responsável. É imperativo buscar melhores indicadores de pobreza, especialmente no contexto rural, para podermos tomar decisões informadas que considerem tanto os indicadores quanto a floresta e as pessoas que dependem dela.
